# Benign proliferating pilar tumor excised with Slow Mohs surgery: A case report

**DOI:** 10.1177/2050313X231213928

**Published:** 2023-11-22

**Authors:** Krystal Stewart, Doha Itani, Andrew Mulherin, Robert Hayes

**Affiliations:** 1 Department of Medicine, Dalhousie University, Saint John, NB, Canada; 2 Department of Pathology and Laboratory Medicine, Dalhousie University, Saint John, NB, Canada; 3 Division of Plastic and Reconstructive Surgery, Dalhousie University, Saint John, NB, Canada; 4 Division of Clinical Dermatology and Cutaneous Science, Dalhousie University, Saint John, NB, Canada

**Keywords:** Proliferating pilar tumor, proliferating trichilemmal tumor, benign, Slow Mohs, Mohs micrographic surgery

## Abstract

Proliferating pilar tumors are rare, benign, exophytic neoplasms, which can closely resemble a squamous cell carcinoma. We describe a patient with a large benign exophytic tumor on the scalp that had been slowly growing over 10 years. While this class of benign follicular tumors is rare, the standard of care is typically excision with clear histologic margins. In this case, this large scalp tumor was surgically excised with clear margins/permanent section margin control using “Slow Mohs” technique, with subsequent repair using a skin substitute dressing, followed by a delayed skin graft.

## Introduction

Proliferating pilar tumor (PTT) is a rare, benign, exophytic tumor that may sporadically proliferate from a preexisting trichilemmal cyst.^1^ These tumors can also arise de novo and they may clinically resemble a squamous cell carcinoma.^2^ PTTs are benign follicular tumors that are classified under infundibular and/or isthmic differentiation, where the infundibular epithelium is indistinguishable from adjacent epidermis.^2^ It is characterized histologically as a partially cystic and solid lesion with increased keratinocyte size, abrupt keratinization, and the absence of a granular layer.^2^ They are more commonly observed in women over the age of 40, and 90% of cases are located on the scalp.^3^ Rarely, PTTs may differentiate into a malignant neoplasm, with the potential for metastasis.^1,2^ Wide local excision is the standard of care due to the potential for recurrence and risk of malignant transformation.^1^ As these tumors have the potential to grow large and lead to a significant surgical defect, the use of excision with margin control, such as Slow Mohs micrographic surgery technique, has the potential to be tissue-sparing.

## Case report

**Figure 1.**
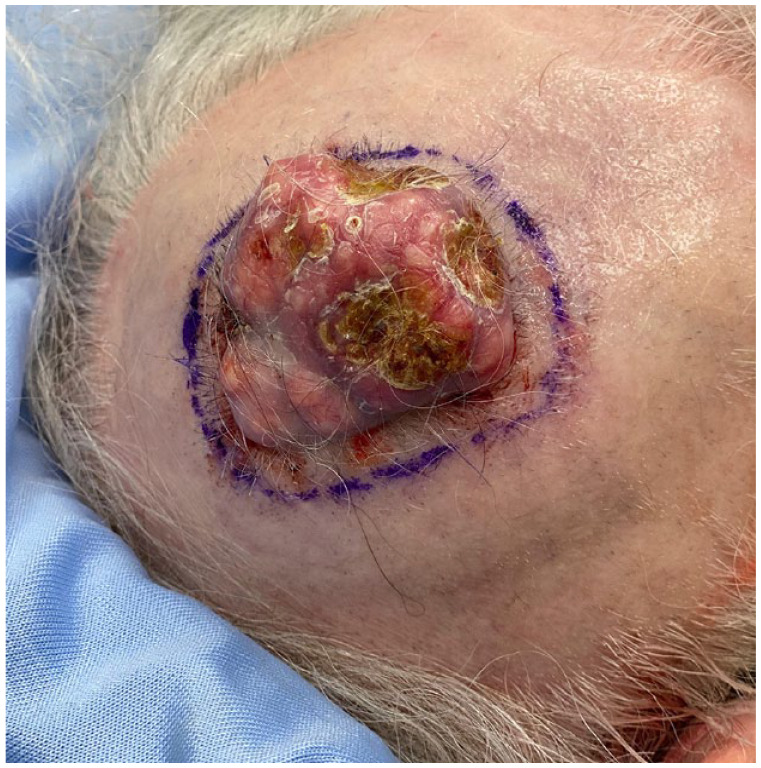
Pre-excision of PPT.

**Figure 2.**
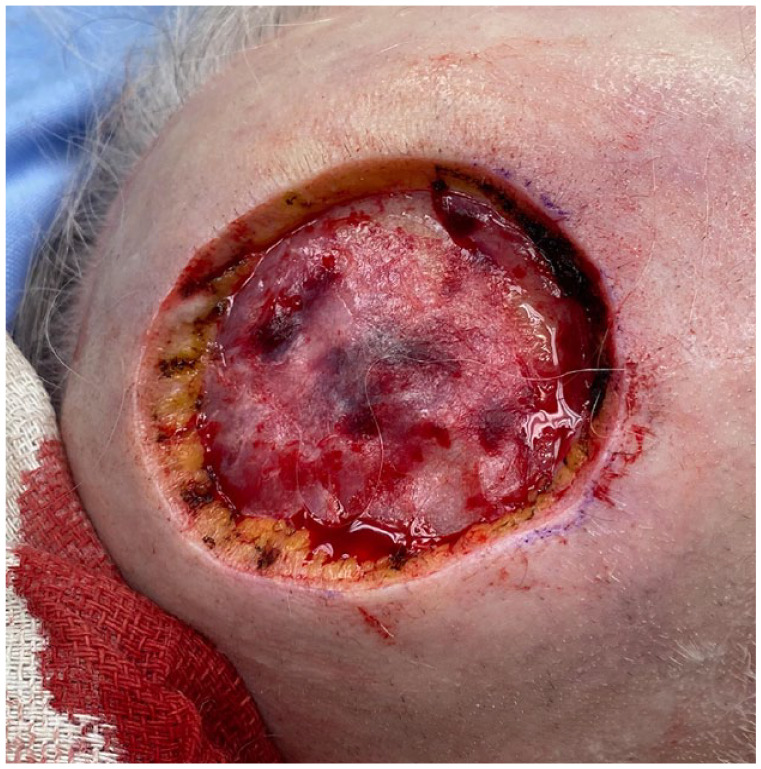
Defect post-excision.

A healthy 77-year-old woman presented with a 5-cm pink, dome-shaped, eroded exophytic tumor, over her right parietal scalp that had been slowly growing for 10 years (Figure 1). The tumor was excised under local anesthesia, using a Slow Mohs technique with permanent section margin control. The entire tumor was resected en bloc, with 8-mm lateral margins, and deep margins down to pericranium (Figure 2). A Xeroform (Covidien, Mansfield, MA) bolster dressing was secured over the wound and the patient was referred to a plastic and reconstructive surgeon to discuss options for closure.

**Figure 3.**
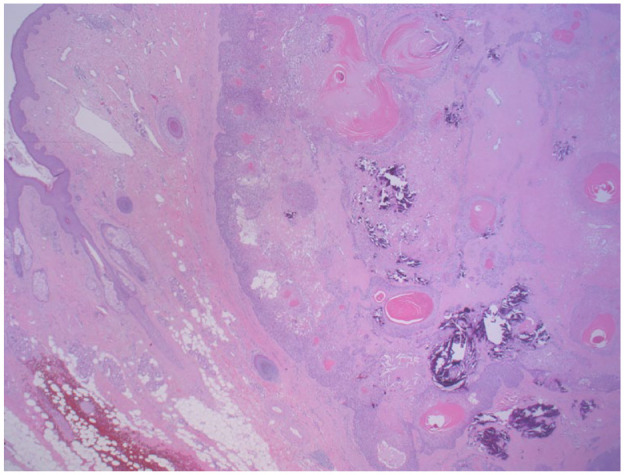
Hematoxylin and eosin stain showing the smooth pushing edge of proliferating pilar tumor at 20× magnification.

**Figure 4.**
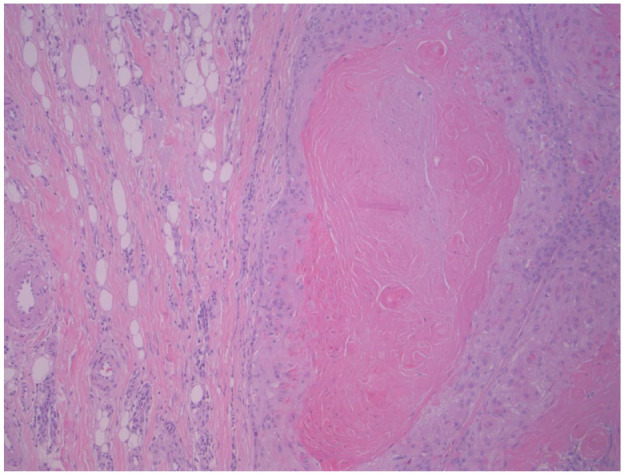
Hematoxylin and eosin stain showing the abrupt keratinization of proliferating pilar tumor at 200× magnification.

Permanent section pathology revealed a keratinizing tumor with symmetrical architecture and a circumscribed, pushing border. There were multiple areas of abrupt keratinization, cystic ulceration, and calcification, consistent with a benign proliferating pilar (trichilemmal) tumor (Figures 3 and 4).

**Figure 5.**
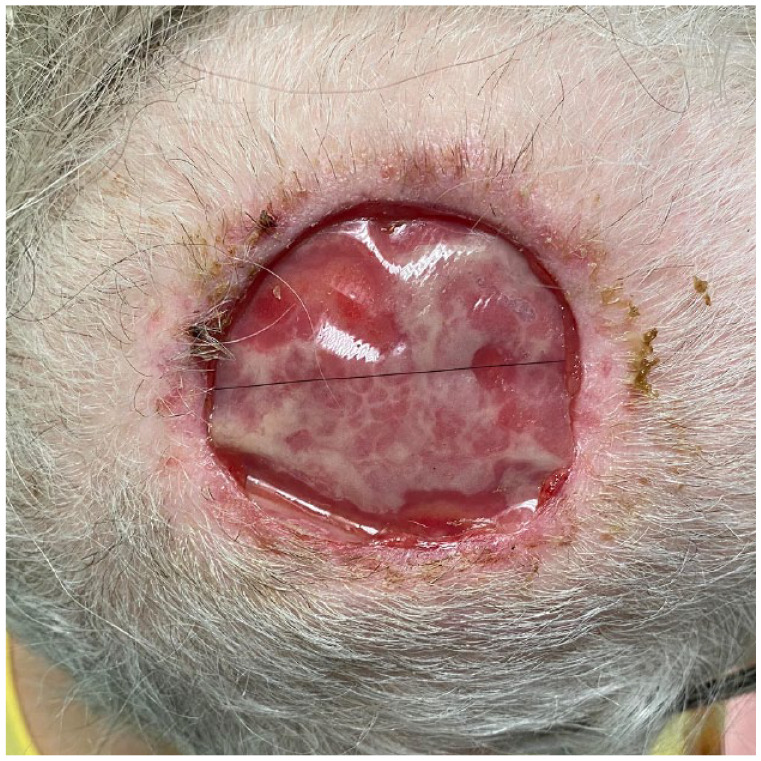
Wound bed with Integra application.

**Figure 6.**
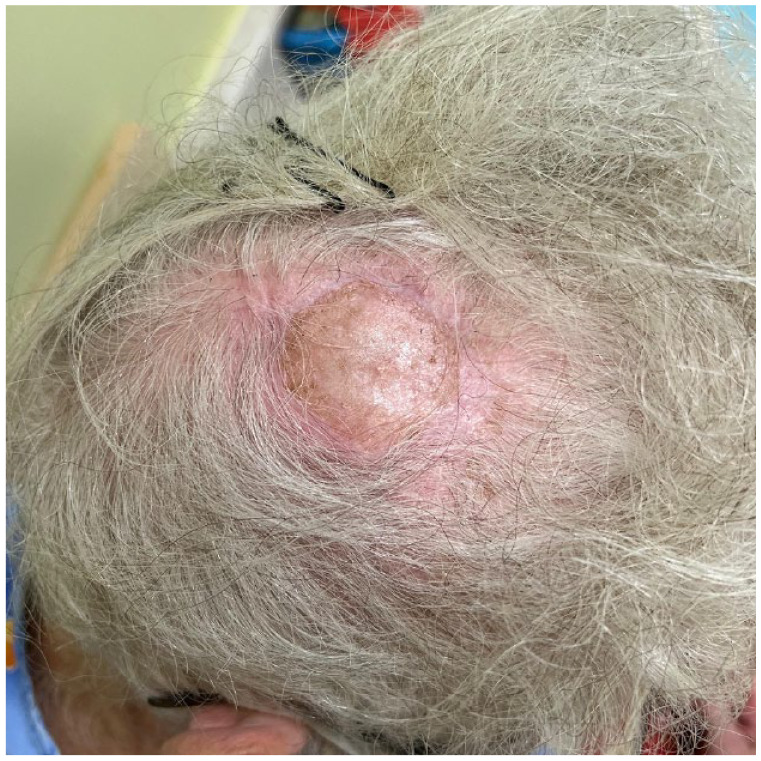
Healthy scar at 7 months’ follow-up.

Two weeks later, examination revealed desiccated periosteum with no granulation of the wound bed. The outer cranial table was burred to spongy bone (diploë) and covered with a collagen-glycosaminoglycan biodegradable matrix (Integra) (Integra Life Sciences, Princeton, NJ) (Figure 5). After 6 weeks, once the biodegradable matrix had fully vascularized, a split thickness skin graft was taken from the thigh and applied to the granulated wound bed. A tie-over bolster was then applied for 7 days. The skin graft adhered well and healed without any complications. The patient was happy with the final functional and cosmetic result, and there was no sign of recurrence of PTT at 7 months’ follow-up (Figure 6).

## Discussion

Most cases of benign PTTs located on the scalp involve patients who tend to be female and over the age of 55 years, with a few reports of patients being in their 20s.^4–15^ The average size of the tumors was approximately 5 cm in diameter.^4–15^

This case report is congruent with the typical patient profile for a benign PTT. Slow Mohs technique with meticulous, mapped, and oriented permanent section margin control, was used to excise the tumor, with histologic examination by a pathologist. A wide excision (with variable margins) is the standard procedure for removing benign PTTs according to the current literature, with over 75% of the case reports opting for this method.^4–9,11–14^ There are no formal recommendations for margin size and depth in removal of either benign or malignant PTTs. Consequently, the margin size among these cases tended to be arbitrary and considered to be surgeon dependent, with some stating 1 cm is sufficient while other articles proposed that 2 cm is ideal as a precaution to avoid local recurrence.^4–9,11–14^ Benign PTTs typically grow in size between 2 and 10 cm in diameter, but they have the potential of growing as big as 25 cm; by adding an additional margin size this will only create a larger defect and thus a more difficult closure.^3^ In one report, radiotherapy was used to clear several small nodules on the scalp that were considered unresectable, after previous surgical removal.^10^

Mohs micrographic surgery utilizes precise frozen section histologic margin control to remove skin cancers while maximizing tissue preservation.^16^ “Slow Mohs” technique with mapped permanent section margin control was chosen for this large scalp PTT to ensure clear margins, preserve healthy tissue, and shorten the procedural time for the patient. There are few case reports of Mohs surgery for PTT in the literature. Tierney et al.^15^ described Mohs surgery for a PTT, but due to the large defect size, a tissue expander was used for reconstruction. Alarcón et al.^5^ described two cases of PTT treated with Mohs surgery, both with free margins after the second stage.

Complete excision of PTT is essential to avoid tumor recurrence. Rarely, PTT has the potential for malignant transformation, with ulceration, bleeding, rapid growth, infiltration into the outer cranial table, and potential metastases to the cervical lymph nodes.^4^ Mohs micrographic surgery with frozen sections, or a modified Slow Mohs technique with permanent sections, has the advantage of histologic margin control for the removal of scalp PTT. Mohs surgery preserves functionally and aesthetically important scalp tissue, by examining all histologic margins, identifying any potential subclinical extensions of PTT, thereby reducing the risk of PTT recurrence.^16^

## References

[1] Alshaalan Z Patel P Routt E , et al. Proliferating pilar tumor: two cases and a review of the literature. J Drugs Dermatol 2021; 20(12): 1346–1348. 34898151 10.36849/jdd.5978

[2] Tellechea O Cardoso JC Reis JP , et al. Benign follicular tumors. An Bras Dermatol 2015; 90: 780–798. 26734858 10.1590/abd1806-4841.20154114 PMC4689065

[3] Requena L Crowson AN Michal M , et al. Proliferating trichilemmal tumour. In: Elder D Massi D Scolyer R , et al. (eds.) WHO classification of skin tumours. 4th ed. Lyon, France: International Agency for Research on Cancer, 2018, pp. 196–197.

[4] Benslimane Kamal I Hali F Marnissi F , et al. The proliferating and malignant proliferating trichilemmal cyst: an anatomo-clinical study of three cases. Skin Appendage Disord 2021; 8(2): 161–164. 35419418 10.1159/000518354 PMC8928208

[5] Alarcón Pérez CE Gómez Ángulo D Olmos Pérez M , et al. Management of 3 proliferating pilar tumors: definition, differential diagnosis, and treatment options. Actas Dermosifiliogr (Engl Ed) 2019; 110(10): 850–854. 31151667 10.1016/j.ad.2018.08.010

[6] Alam K Gupta K Maheshwari V , et al. A large proliferating trichilemmal cyst masquerading as squamous cell carcinoma. Indian J Dermatol 2015; 60(1): 104. 10.4103/0019-5154.147854 PMC4318032 25657426

[7] Garetto F Morozzo G Morozzo U , et al. Recurrent proliferating trichilemmal cyst of the scalp. G Ital Dermatol Venereol 2018; 153(1): 107–110. 26426376 10.23736/S0392-0488.17.04882-9

[8] Naciri I Hassam B . [Exophytic tumor of the scalp]. Pan Afr Med J 2017; 28: 45. 29184598 10.11604/pamj.2017.28.45.13535 PMC5697991

[9] Sethi S Singh UR . Proliferating trichilemmal cyst: report of two cases, one benign and the other malignant. J Dermatol 2002; 29(4): 214–220. 12027086 10.1111/j.1346-8138.2002.tb00252.x

[10] Parambeth HK Udhayam N Agarwal S , et al. A large helmet-shaped proliferating trichilemmal tumor of the scalp: is definitive radiotherapy the treatment? A case report. J Egypt Natl Canc Inst 2019; 31(1): 7. 32372190 10.1186/s43046-019-0007-y PMC13317719

[11] Sharma R Verma P Yadav P , et al. Proliferating trichilemmal tumor of scalp: benign or malignant, a dilemma. J Cutan Aesthet Surg 2012; 5(3): 213–215. 23112524 10.4103/0974-2077.101394 PMC3483585

[12] Warner TF . Proliferating pilar cyst with spindle cell component. J Cutan Pathol 1979; 6(4): 310–316. 500878 10.1111/j.1600-0560.1979.tb01139.x

[13] Anolik R Firoz B Walters RF , et al. Proliferating trichilemmal cyst with focal calcification. Dermatol Online J 2008; 14(10): 25. 19061624

[14] Khoja AA Yan B Lee SJ , et al. Proliferating tricholemmal tumour: clinicopathological aspects of a case. Singapore Med J 2011; 52(12): e255–e257. 22159947

[15] Tierney E Ochoa MT Rudkin G , et al. Mohs’ micrographic surgery of a proliferating trichilemmal tumor in a young black man. Dermatol Surg 2005; 31(3): 359–363. 15841643 10.1111/j.1524-4725.2005.31090

[16] Prickett KA Ramsey ML . Mohs micrographic surgery. In: StatPearls. Treasure Island, FL: StatPearls Publishing, 2023. 28722863

